# From Reddit to Wall Street: the role of committed minorities in financial collective action

**DOI:** 10.1098/rsos.211488

**Published:** 2022-04-06

**Authors:** Lorenzo Lucchini, Luca Maria Aiello, Laura Alessandretti, Gianmarco De Francisci Morales, Michele Starnini, Andrea Baronchelli

**Affiliations:** ^1^ Bocconi University, Milano 20100, Italy; ^2^ FBK—Fondazione Bruno Kessler, Trento 38123, Italy; ^3^ IT University of Copenhagen, Kobenhavn, Denmark; ^4^ Technical University of Denmark, Kgs. Lyngby DK-2800, Denmark; ^5^ ISI Foundation, via Chisola 5, Torino 10126, Italy; ^6^ Department of Mathematics, City University of London, London EC1V 0HB, UK; ^7^ The Alan Turing Institute, British Library, 96 Euston Road, London NW1 2DB, UK; ^8^ UCL Centre for Blockchain Technologies, University College London, London, UK

**Keywords:** collective action, coordination dynamics, networks‌, social media

## Abstract

In January 2021, retail investors coordinated on Reddit to target short-selling activity by hedge funds on GameStop shares, causing a surge in the share price and triggering significant losses for the funds involved. Such an effective collective action was unprecedented in finance, and its dynamics remain unclear. Here, we analyse Reddit and financial data and rationalize the events based on recent findings describing how a small fraction of committed individuals may trigger behavioural cascades. First, we operationalize the concept of individual commitment in financial discussions. Second, we show that the increase of commitment within Reddit pre-dated the initial surge in price. Third, we reveal that initial committed users occupied a central position in the network of Reddit conversations. Finally, we show that the social identity of the broader Reddit community grew as the collective action unfolded. These findings shed light on financial collective action, as several observers anticipate it will grow in importance.

## 1. Introduction

In January 2021, the GameStop shares traded on the New York Stock Exchange experienced a classic ‘short squeeze’ [1,2]. As the price sharply jumped higher, traders who had bet that its price would fall (i.e. who ‘shorted’ it) were forced to buy it in order to prevent even greater losses, thus further promoting the price rally [2–4]. Victims of the squeeze were professional hedge funds, and particularly Melvin Capital Management who lost 53% of its investments for a total estimated 4.5 billion USD [5]. The short squeeze was initially and primarily triggered by users of the subreddit r/wallstreetbets (WSB), a popular Internet forum on the social news website Reddit, who managed to translate online discussions into a highly coordinated financial operation.

These events garnered huge attention from the media, professionals and financial authorities. Notably, the US Treasury Secretary Janet Yellen convened a meeting of financial regulators including the heads of the Securities and Exchange Commission, Federal Reserve, Federal Reserve Bank of New York and the Commodity Futures Trading Commission to examine the GameStop squeeze [6]. Cindicator Capital, a fund specialized in digital assets, published a hiring call for a sentiment trader (i.e. a trader trying to gain an advantage by reading the signals about how other investors are feeling about a particular stock) with 3 years of active trading experience and having been a member of WallStreetBets for more than a year with karma—a Reddit measure of ‘how much good the user has done’ for the community—of more than 1000 [7]. Finally, the House Committee on Financial Services of the US Congress held a hearing titled *Game Stopped? Who Wins and Loses When Short Sellers, Social Media, and Retail Investors Collide* to discuss the events [8]. They called as witness Reddit user Keith Gill, known as u/DeepFuckingValue on WSB, who had a central role in triggering the collective action. At the hearing, committee members expressed concern with respect to gamification of investment [9], encouraged by trading platforms such as Robinhood, largely adopted by retail investors due to low commissions. However, how the coordination on WSB took place in the first place remains unclear, despite the importance of clarifying this mechanism in order to assess risks and devise regulations.

In this paper, we analyse discussions on WSB from 27 November 2020 to 3 February 2021 (table 1) and investigate how they translated into collective action before and during the squeeze that was initiated on 22 January and lasted until 2 February. Motivated by recent theoretical [10,11] and experimental [12] evidence that minorities of committed individuals may mobilize large fractions of a population [10,13–15] even when they are extremely small [16], we investigate whether committed users on WSB had a role in triggering the collective action. To this aim, we operationalize the commitment of a user as an exhibited proof that the user has financial stakes in the asset.

**Table 1.** Key events relevant to the GameStop (GME) short squeeze.

	event	date	description
*a*	GME earnings	8 Dec 2020	GME earning reports revealed a 257% increase in e-commerce revenues
*b*	new board	11 Jan 2021	GME announced a renewed Board of Directors, which included experts in e-commerce.
*c*	Citron prediction	19 Jan 2021	Citron Research, a popular stock commentary website, published a piece predicting the value of the GME stock would decrease. Citron Research is managed by Andrew Edward Left, a financial analyst and renowned short seller.
*d*	Elon Musk’s tweet	26 Jan 2021	Business magnate Elon Musk tweeted ‘Gamestonk!!’ along with a link to WSB.

We show that a sustained commitment activity systematically pre-dates the increase of GameStop share returns, while simple measures of public attention towards the phenomenon cannot predict the share increase. Additionally, we also show that the success of the squeeze operation determines a growth of the social identity of WSB participants, despite the continuous flow of new users into the group. Finally, we find that users who committed early occupy a central position in the discussion network, as reconstructed by WSB posts and comments, during the weeks preceding the stock price surge, while more peripheral users show commitment only in the last phases of the saga.

### 1.1. The GameStop saga

GameStop (GME) is a US video game retailer which was at the centre of the short squeeze in January 2021. The timeline of the events around the squeeze is summarized in table 1, and it unfolded as follows. In 2019, Reddit user u/DeepFuckingValue entered a long position on GME, i.e. he bought shares of the GME stock, and started sharing regular updates in WSB. On 27 October 2020, Reddit user u/Stonksflyingup shared a video explaining how a short position held by Melvin Capital, a hedge fund, could be used to trigger a short squeeze. On 11 January 2021, GME announced a renewed Board of Directors, which included experts in e-commerce. This move was widely regarded as positive for the company, and sparked some initial chatter on WSB. On 19 January, Citron Research (an investment website focused on shorting stocks) released a prediction that GME’s stock price would decrease rapidly. On 22 January, users of WSB initiated the short squeeze. By 26 January, the stock price increased more than 600%, and its trading was halted several times due to its high volatility. On that same date, business magnate Elon Musk tweeted ‘Gamestonk!!’ along with a link to WSB. On 28 January, GME reached its all-time intra-day highest price, and more than 1 million of its shares were deemed failed-to-deliver, which sealed the success of the squeeze. A failure to deliver is the inability of a party to deliver a tradable asset, or meet a contractual obligation; a typical example is the failure to deliver shares as part of a short transaction. On 28 January, the financial service company Robinhood, whose trading application was popular among WSB users, halted all the purchases of GME stocks. On 1 and 2 February, the stock price declined substantially.

By the end of January 2021, Melvin Capital, which had heavily shorted GameStop, declared to have covered its short position (i.e. closed it by buying the underlying stock). As a result, it lost 30% of its value since the start of 2021, and suffered a loss of 53% of its investments, i.e. more than 4 billion USD.

### 1.2. The r/wallstreetbets ecosystem

Reddit is a public discussion website structured in an ever-growing set of independent *subreddits* dedicated to a broad range of topics. Users can submit new *posts* to any subreddit, and other users can add *comments* to existing posts or comments, thus creating nested conversation threads. One such subreddit is r/wallstreetbets (WSB), a forum for investors and traders on Reddit, which self-describes with the tagline ‘Like 4chan with a Bloomberg terminal’. It is dedicated to high-risk trades involving derivative financial products (e.g. options and futures, often leveraged), and is thus not targeted to the beginner investor, but to somewhat experienced retail traders. Created in 2012, as of June 2021, it counts more than 10 million subscribers (self-proclaimed ‘degenerates’, but also known as ‘autists’, ‘retards’ and ‘apes’, depending on the type of information shared on the subreddit). As is clear from its description so far, the WSB community is known for its profane and juvenile humour, and has a well-defined identity reinforced also by the common use of jargon (e.g. ‘stonks’ for stocks, ‘tendies’ for profits, and ‘diamond hands’ or ‘paper hands’ for people that hold stocks through turbulent times or sell them at the first loss, respectively). The popularity of this forum has increased in recent years (since 2017 especially), possibly also due to the widespread adoption of no-commission brokers and mobile online trading platforms such as robinhood.com.

The topics of discussion on the forum are varied, but there are some common patterns of behaviours which are also described in the FAQ [17]. When submitting a post, a user can apply a category tag called ‘flair’, which serves as an indication of its content. The allowed flairs, together with a short description, are reported in table 2. The community takes flairs seriously and strictly enforces them (e.g. the FAQ report that misusing important flairs can lead to getting permanently banned). It is thus very common to find posts containing screenshots of an open position on a risky bet tagged with a YOLO flair, all interspersed with unhinged humorous posts and memes.

**Table 2.** Flairs allowed on r/wallstree tbets and their meaning as per the subreddit guidelines.

flair	meaning
YOLO (You Only Live Once)	YOLO flair is for dank trades only. The minimum value at risk must be at least $10 000 in options, or $25 000 in equity.
DD (due diligence)	The research you have done on a specific company/sector/trade idea. This is a high effort text post. It should include sources and citations. It should be a long post and not just a link to a submission.
discussion	An idea or article that you would like to talk about. Needs to be more involved than ‘up or down today?’
gain	Use this flair to show off a solid winning trade. Minimum gain is $2500 for options, $10 000 for shares. You must show or explain your trade. If you have to say something like ‘position in comments’ then it’s a bad screenshot.
loss	Show off a brutal, crushing loss. Minimum loss $2500 for options, $10 000 for shares. You must show or explain your trade. If you have to say something like ‘position in comments’ then it’s a bad screenshot.

The discussion within the subreddit follows a simple post-comment dynamic, where each post separately grows its multi-level comment tree. Each interaction, it being a post or a comment, can additionally receive ‘upvotes’ and ‘downvotes’. While ‘upvoting’ or ‘downvoting’ represents a typical ‘slacktivist’ practice for anonymously expressing one’s position, other users can also choose to ‘award’ prizes to more emphatically recognize a post or comment.

## 2. Results

### 2.1. Collective attention, commitment and identity on WSB

In this work, we mark as *commitment events* the posts in which the authors provided proof of their financial stake in the GameStop stock. Commitment is established by including specific flair categories (YOLO, gain and loss) and by using computer vision techniques to identify relevant screenshots taken from any online trading applications and posted on WSB (see Methods). We retrieve 433 785 screenshots and classify 5947 of those as commitment proofs. In total, we identify 36 128 events of commitment by 30 133 users.

**Figure 1.**
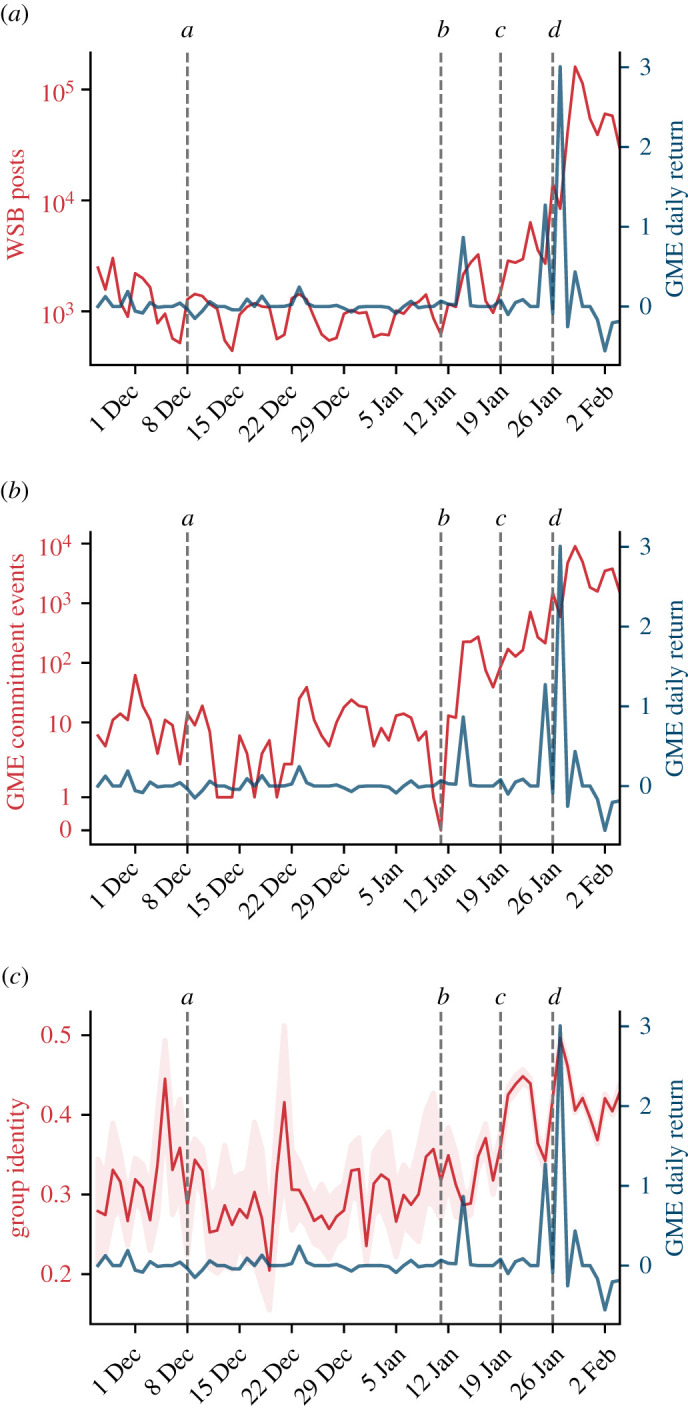
GME stock returns compared with: (*a*) number of posts submitted on WSB; (*b*) number of posts on WSB that showed financial commitment; (*c*) level of group identity (shaded areas corresponds to 2 s.e. of the daily average).

Figure 1 compares the daily returns of GME (the per cent increase of price compared with the previous day) with three quantities calculated over time on a daily basis: (i) activity within the community measured as the number of posts submitted on WSB; (ii) number of posts on WSB that showed financial commitment towards GME stocks; and (iii) level of group identity signalled by language markers in WSB submissions about GME.

The posting activity in the WSB community is characterized by a weekly periodicity that endures stably until the announcement of a renewed Board of Directors (event *b*). After the first considerable increase in the stock price on 14 January (+57%), the activity grows noticeably. Posting activity raises exponentially after the second price spike on 26 January (+92%) and it culminates on 28 January, 2 days after the stock evaluation reached its maximum. Public attention to the GME phenomenon spreads far beyond the boundaries of Reddit. The number of GME-related tweets follows a similar exponential growth starting on 27 January after Elon Musk’s endorsement (event *d*) and peaking on 28 January (electronic supplementary material, figure S1*c*). The growing interest on Twitter matches the explosive growth in the number of new subscribers to the WSB subreddit (electronic supplementary material, figure S1*a*,*b*). Overall, these results support three conclusions. First, collective attention towards GME follows the asset price growth with a delay. Second, despite the collective action being designed and coordinated on Reddit, wide interest was expressed on other social media as well. Last, not only the discussion originated from Reddit gradually attracted the attention of larger crowds to the topic but it also engaged those crowds to the point of attracting new users to the original source of the discussion—the WSB subreddit.

The evolution of commitment over time differs considerably from the growth of collective attention. Figure 1*b* shows the number of daily commitment events measured by counting ‘gain’, ‘loss’, ‘YOLO’ posts (i.e. posts with one of these flairs) and the screenshots that WSB users submitted as proof of stake (see Methods for details). Before the new board of directors was announced (event *b*), WSB users uploaded a few dozens of commitment posts per day. The number of commitment posts increases 10-fold on the day of the first price spike and keeps growing steadily afterwards. For 11 days, between the first price spike on 14 January until the next spike on 25 January, such increase in commitment takes place in the absence of any growth in financial returns. In summary, commitment pre-dates price surges and is sustained also in the absence of gains.

The presence of commitment in the absence of returns raises the question of whether commitment was supported by other processes endogenous to the WSB community. A recent ethnographic study found that active members of WSB use shared linguistic markers and reciprocation of custom awards to express and reinforce the community’s sense of identity [18]. Identity is a shared sense of belonging to a group [19] that can influence inter-group behaviour [20], not least by fostering cooperation [21,22]. Identity is often signalled explicitly through symbols [23] or language cues [24]. To measure the group identity in GME-related submissions, we used a validated indicator of group identity [25]: for each submission, we calculated the fraction of the first person pronouns that are plural (i.e. the number of references to *we* over the sum of the number of references to *we* and *I*), and averaged those fractions across all the submissions of a given day (more details in Methods). Figure 1*c* shows the group identity expressed by GME-related submissions within the WSB community. The signal oscillates heavily until mid-January, due to the relatively low number of submissions. As the number of submissions increases, we detect two peaks. The first peak follows the market analysis from Citron Research (event *c*) that forecast a drop in GME stock price and antagonized the members of the WSB community by referring to them as ‘suckers at this poker game’. This finding is in agreement with the theoretical expectation of community identity being created during processes of struggle between social groups [26]—in this case, between WSB and its detractors. The second peak matches the maximum increase of the stock price, and it is probably caused by the acknowledgement of collective success in performing the short squeeze. In short, we find that expressions of identity emerged concurrently with the increase of commitment and might have played a role in sustaining it, but identity is unlikely to be the origin of the collective action.

### 2.2. Commitment and reach of core versus peripheral authors

**Figure 2.**
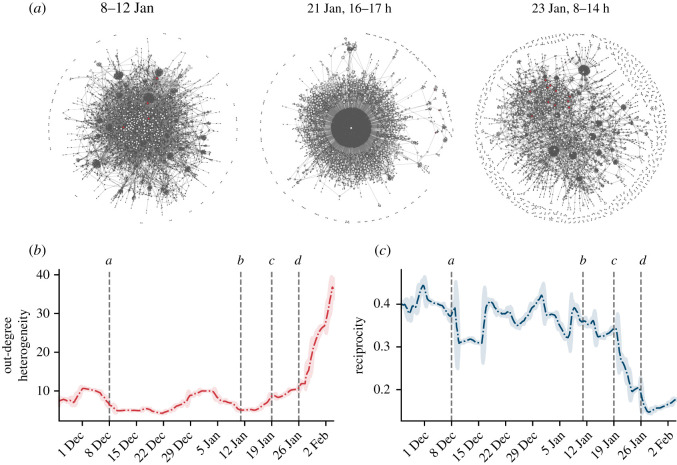
The evolution of the GME discussion network. (*a*) Three snapshots of GME discussion networks, reconstructed in different time windows with the same number of nodes, *N* = 3000. Nodes are WSB users, coloured according to whether they posted a commitment submission (red) or not (grey). The size of nodes is inversely proportional to their *D*-shell, i.e. nodes belonging to the core are bigger than peripheral nodes. A link exists between two nodes if one of the two replied to the other at least once. (*b*,*c*) Two key topological features of networks reconstructed over a rolling time window of 7 days (see electronic supplementary material, figure S6 for more results using different windows): (*b*) The heterogeneity of the degree distribution *κ*, defined as *κ* ≡ 〈*k*^2^〉/〈*k*〉^2^, where 〈*k*〉 and 〈*k*^2^〉 are the first and second moments of the degree distribution, and *n*-th moment is $\langle k^{n}\rangle \equiv \sum_{i}^{N}k_{i}^{n}/N$ [27]. (*c*) The average network reciprocity. Shaded areas represent 2 s.d. of the network metrics aggregated on a daily rolling window basis.

The sustained flow of commitment events during the weeks preceding stock price surge indicates the presence of a minority of committed users. As the interaction between the committed minority and the rest of the community is crucial to the success of a collective action [10,13,14], we study the dynamics of social interactions between committed individuals and other WSB users. These interactions occur over a rapidly evolving social network. Figure 2*a* shows a few snapshots of the network of replies over time. In these networks, users are connected if they submitted a comment in reply to the post or comment of another during the time-span considered. As new users join, the number of small disconnected components in the network increases (see electronic supplementary material, figure S4), while the connected component tends to cluster around few popular discussion threads (especially the so-called *daily megathreads* [18]) created with the purpose of summarizing the events of the day and planning future actions. The structural transformation of the network happens abruptly rather than gradually.

We quantify this structural change by reconstructing networks over a rolling time window of 7 days, and looking at the evolution of two key topological quantities of these networks in time. First, we observe that the heterogeneity of the distribution of the nodes’ out-degree (i.e. the number of different users each user replies to) [27] increases threefold in the span of 20 days after event *c*, thus reflecting the simultaneous emergence of super-hubs of discussion together with users engaging only in isolated interactions. Second, the direct reciprocity of interaction (i.e. the fraction of replies that are reciprocated within the time window considered) gets roughly halved in the same time span (figure 2*c*). This signal, combined with the increase in expressions of group identity (figure 1*c*), is compatible with the emergence of generalized reciprocity [21], a norm according to which individual messages are not expected to receive direct responses; comments are not perceived as pieces of a conversation but rather as contributions to a collective discussion from which everyone benefits. Indeed, the increase in size of the discussion naturally leads to a more fragmented conversation where direct replies are less frequent, and thus to a decrease in reciprocity.

The complex and dynamic nature of the social network raises the question of what is the typical position of committed users in the network, and whether this position changes over time. To answer this question, we operationalize the notion of network position with the concept of *D*_*k*,*l*_-*core shell* [28,29]: the set of nodes in which every node is connected with other members of the set with at least *k* outgoing links and *l* incoming ones. This measure is a good indicator of a node’s centrality because it directly gauges embeddedness (the density of connections around it), and it is a good proxy for reachability (how quickly it can be reached from any other node of the network).

For each temporal slice of the network, we perform its *D*_*k*,*l*_-core decomposition (see Methods), and we measure the level of commitment exhibited in each *D*_*k*,*l*=*k*_-core shell. Borrowing from previous work [30], we estimate the potential influence that commitment events have on the community at large by measuring not only the volume of commitment events in a shell but also the number of people that these events *reach*—namely the number of WSB members who commented on a post that is submitted by a committed node in that shell.

**Figure 3.**
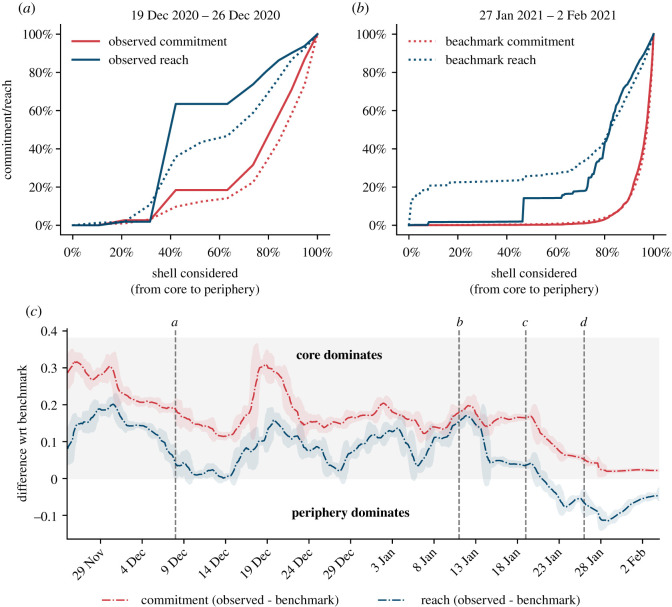
The evolution of commitment and reach. (*a*,*b*) Fraction of observed commitment activity (red line) and reach (blue line) produced by nodes belonging to an increasingly large fraction of *D*-core shells (from core to periphery). Curves constructed using the observed data (filled lines) are compared with those obtained for the benchmark model (dashed lines). Results are shown for two network slices, the first constructed between 19 December and 26 December (*a*), the second between 27 January and 2 February (*b*). (*c*) Average difference between the area below the observed curve and the area below the benchmark curve over time, for commitment (red dashed-dotted line) and reach (blue dashed-dotted line). Shaded areas corresponds to the 2 s.d. area computed for each slice. Dashed vertical lines indicate relevant events (table 1). For values of difference larger than zero (grey shaded area), activity is concentrated in the core of the network, relative to the benchmark model. Networks are constructed using a sliding window of 7 days.

Figure 3 shows how commitment activity and its reach are distributed between users in the core of the network (high *D*-core shells) and peripheral users (low *D*-core shells), as a function of time. First, in figure 3*a*,*b*, we show the fraction of commitment activity and reach that are generated by nodes belonging to an increasingly large number of *D*-core shells, taken from the core to the periphery. To disentangle the effect of the network’s evolution from the distribution of commitment and reach on the network, and meaningfully compare commitment distribution over networks reconstructed in different periods, we contrast the observed commitment activity and its reach with a null model benchmark which preserves the network’s topology but randomizes the distribution of commitment over it (see Methods). In figure 3*a*,*b*, the curve being higher (lower) than the benchmark indicates that commitment volume or reach are generated predominantly by nodes in the core (periphery) of the network. For example, in the network of interactions between 19 December and 26 December, central nodes are those who pledge more commitment to the GME cause (figure 3*a*); on the contrary, when considering interactions between 27 January and 2 February, commitment comes mostly from peripheral actors (figure 3*b*).

To get a comprehensive picture of the coreness of committed users over time, we measure the difference between the area below the observed curve and the area below the benchmark curve at a given temporal slice, for all the slices computed on networks reconstructed by using a rolling time window of 7 days. Results are robust to the slicing strategy chosen for constructing the networks (see electronic supplementary material, §A.6, figures S7 and S8), to the size of the slices (see electronic supplementary material, §A.7, figures S9 and S10), and to *D*-core selection (see electronic supplementary material, §A.8, figures S11 and S12). Figure 3*c* shows the value of such difference as a function of time. Relative to the benchmark model, both commitment and reach are concentrated in the network’s core until event *c* (19 January). From that moment onward, the commitment activity obtains a larger reach within the periphery. While always remaining more concentrated in the core, the commitment activity spreads more and more towards the periphery following event *c*. Therefore, the committed minority which may have triggered the first price increase in the GME stock is formed by central users in the discussion network unfolding on WSB. Only in the last phases of the collective action, when the price has already increased considerably, peripheral users step in and show commitment, which reaches more peripheral peers.

## 3. Discussion

In this paper, we showed that the collective action originated on Reddit and culminated in the successful short squeeze of GameStop shares was driven by a small number of committed individuals. We operationalized financial commitment on Reddit as providing proof of stakes in a given asset, often in the form of a screenshot. We then showed that events of commitment pre-dated the initial surge in price, which in turn attracted more participants to the GameStop discussion and thus triggered new events of commitment. Finally, we described how initial committed users were part of the core of the network of Reddit conversations, and that the social identity of the broader group of Reddit users grew as the collective action unfolds.

Our study focused on a single, unprecedented, event of financial collective action. While this is certainly a limitation, as more events would allow us to corroborate or falsify our findings, a prompt investigation of the GameStop events was in order. The main contribution of our paper is framing a *novel* collective coordination phenomenon of unprecedented nature within a well-established sociological framework. While we do not explicitly validate any hypothesis, we discard other possibly competing explanations, such as social identity playing a role in the coordination dynamics.

To this aim, we leverage well-established methods from machine learning and network science, to introduce a novel approach to study financial coordination on social media. Specifically: (i) we operationalize financial commitment of Reddit users by classifying their posts (we produce a new ground truth and train a new classifier to do so) and (ii) we analyse the position of committed user in the discussion network and introduce a new network null model to assess fairly any core-periphery shift over time. While the way in which we classify commitment is specific to an individual event, the methods we propose generalize to any platforms in which commitment and social interactions can be measured.

The events that unfolded over the course of the few weeks that we analysed in our study caused sustained effects on the market. Seven months later (at the time of writing this manuscript), the value of the GameStop stocks had risen by 1000% compared with the beginning of 2021. The price increase inflicted enormous financial losses to multiple hedge funds, one of which was forced to shut down [31].

The influence of retail investors in equity markets is rapidly growing, and now accounts for almost as much volume as hedge and mutual funds combined [32]. This rise has been mainly driven by the emergence of commission-free trading platforms that offer the possibility to trade fractions of shares, so that users can start trading even with very small amounts. Moreover, these platforms allow investors to use leverage, by buying and selling options and accessing to cheap margin loans from brokerages, in a gamified user experience. This ‘democratization of trading and investing’ is unlikely to disappear any time soon [33], so other financial collective actions might be coordinated in the future, possibly through different social media channels.

In this perspective, beyond the role of committed individuals in promoting the coordinated action, our findings have other potential implications to be tested in future research. (i) The fact that initial committed individuals were part of the core of the Reddit discussions implies that the system may be resilient against adversarial attacks where freshly created ‘committed’ bots try to influence the community. (ii) The finding that identity was not the driver of the collective action but, on the contrary, a by-product of it may imply that successive actions that leverage it might be easier to coordinate. (iii) The change in network structure ensuing from the arrival of new users, who joined the discussion motivated by the initial success of the squeeze, and the corresponding shift of the bulk of commitment and reach from the core to the periphery of the network, highlights the role of the system’s openness and the hierarchies that catalyse a successful collective action.

Taken together, our findings highlight that financial collective action cannot be reduced to the impact of social coordination on financial markets. The effect—and, particularly, the success—of an action have profound consequences on the membership, structure and dynamics of the original group, whose evolution may have in its turn consequences on future actions. Thus, the initial committed individuals trigger a behavioural cascade which is self-sustaining and transforms the group itself. More events and data are needed to clarify this interplay between bottom-up processes of social coordination and financial markets, and this is a direction for future work. Our results represent a first step in this direction, and we anticipate that, as financial collective action is expected to acquire even more importance in the future, they will be of interest to researchers, industry professionals and regulators.

## 4. Methods

### 4.1. Data

We used two main sources of data: the activity on the subreddit r/wallstreetbets and the price of GameStop shares, ticker GME.

Reddit is organized in communities, called *subreddits*, that share a common topic and a specific set of rules. Users subscribe to subreddits, which contribute to the news feed of the user (their home) with new posts. Inside each subreddit, a user can publish *posts* (also called ‘submissions’), or *comment* on other posts and comments, thus creating trees of discussion that grow over time. Users can attach *flairs* to posts: a set of community-defined tags to define the semantic scope of the post, thus facilitating content search and filtering. Users can assign *awards* to posts or comments to recognize their value. Awards are sold by Reddit for money, they come in a variety of types, and some of them reward the recipient with money or perks such as access to exclusive subreddits.

We collected all posts and comments submitted to the r/wallstreetbets subreddit from 1 January 2016 up to the beginning of February 2021. We did so by querying the *Pushshift API* [34], which stores all Reddit activity over time—using the *PMAW* wrapper [35] (see electronic supplementary material, §A.1 for more details). The API returns rich metadata, including the timestamp of submission, the identity of the authors, its text content, and the awards each submission and comment received. In total, we retrieved 1 132 897 posts and 29 566 180 comments submitted to the subreddit by 1 364 080 different authors. We specialize only to posts related to GME by searching for posts containing either in the title or in the text-body the word ‘GME’ or ‘Gamestop’ (lowercase occurrences included) and all the comment trees associated with those submissions. This selected set consists of 129 731 posts and 2 575 742 comments. The period over which our study focuses its attention (from 27 November 2020 to 3 February 2021) includes 99% of the posts and 98% of the comments submitted since 1 January 2016 until 3 February 2021.

We retrieved GameStop daily prices from *Yahoo Finance*, using the Python library *yfinance* [36], and computed the daily price return as the daily relative change, *r*(*t*) = (*p*(*t*)/*p*(*t* − 1)) − 1, where *p*(*t*) is the *Open* price at day *t*.

### 4.2. Quantifying commitment

One of the widely shared norms in the WSB community is to provide proof of one's own financial position when initiating a new discussion about investments [18]. This is commonly achieved by supplementing submissions with screenshots of open positions—typically gains, losses or orders—taken from online trading applications. We used these screenshots to quantify commitment, as they provide a direct way to identify users who had stakes in financial assets. To gather them, we employ two methods: *flairs* and screenshots.

We use three flairs to mark posts containing a proof of position: the *gain* and *loss* flairs mark gains or losses for a minimum of 2500 USD, and the *YOLO* flair indicates investment positions with a minimum value at risk of 10 000 USD. Flair-tagged submissions are moderated and are approved only if a relevant screenshot is attached. While it is mandatory for users to attach investment screenshots to have flairs approved, they can also attach screenshots to their submissions without using any flair.

As we are interested in capturing any signal of commitment, regardless of their magnitude, we resort to machine vision to identify commitment screenshots based on their visual content only. We retrieve all the screenshots attached to any of the submissions in our dataset by querying all URLs terminating with common image extensions (e.g. .png, .jpg). Out of this set, we randomly sample 3745 images and manually inspect them. We mark as *positive* all the screenshots which display gains, losses or orders, and as *negative* all the remaining images, which include a broad variety of content ranging from screenshots of stock prices to memes.

We label 1042 positive examples and 2703 negative examples. We use this set of labelled images to train a supervised model. Among several classifiers available off-the-shelf that we test, the most accurate is a PyTorch [37] implementation of DenseNet [38], a deep neural network architecture designed for image classification. We initialize DenseNet with weights pre-trained on ImageNet [39], a widely used reference dataset of 1.2 million labelled images. We then fine-tune the neural network (i.e. update its weights) by training it further by feeding it 70% of our labelled images. During fine-tuning, we use the Adam optimizer [40] to minimize cross-entropy loss. We then measure the classifier’s performance on the remaining 30% of the examples by using *precision* (the fraction of pictures that the classifier labelled as positive that are actually positive), *recall* (the fraction of positive pictures that the classifier labelled correctly) and F1 score (the harmonic mean between precision and recall). On our validation set, the classifier achieves a precision of 0.85, a recall of 0.73 and an F1 score of 0.77.

We run the classifier on all images from r/wallstreetbets and we merge the posts which contain the images that the classifier marks as positive with the set of flaired posts. In total, following this procedure, we identify 36 128 commitment events. Table 3 shows the number of commitment events divided by event type. Posts can be classified as commitment events by flair type or pictures of a holding position or both at the same time. The ‘unique count’ column shows the contribution to the identification of commitment events uniquely coming from the single commitment type. In electronic supplementary material, figure S2, we show the contributions of each commitment, revealing that commitments from ‘YOLO’ flairs are the dominant ones except for a 3 day period in which the first price surge was followed by a surge in the number of commitment events from ‘Gain’ flairs.

**Table 3.** Commitment events per type. Count column shows the number of posts classified as commitment events because of a ‘YOLO’, ‘gain’, ‘loss’ flair, or a screenshot of commitment identified with our machine vision classifier. Unique count shows the number of posts uniquely classified by the commitment type.

event type	count	unique count	authors	unique authors
YOLO	23 230	21 455	20 107	18 484
gain	7293	6339	6160	5293
loss	2986	2387	2768	2198
pictures	5947	2619	5326	2478

### 4.3. Quantifying identity

To capture linguistic expression of identity, we use two methods. First, we resort to a simple word count approach using linguistic inquiry word count (LIWC). LIWC is a lexicon of words grouped into categories that reflect social processes, emotions and basic functions. It is based on the premise that the words people use provide clues to their psychological states. In particular, the abundant use of words in the LIWC category *we* (i.e. first-person plural subject pronoun) related to the use of words from the LIWC category *I* (i.e. first-person singular subject pronoun) is a validated indicator of group identity [25]. Therefore, we measure identity as the fraction of pronoun *we* against the number of both *we* and *I* pronouns occurring in each submission text body. The results obtained with this particular estimator of identity are robust when compared with two alternative methods, which we discuss in the electronic supplementary material.

### 4.4. Discussion network on WSB

We reconstructed the network of social interactions on r/wallstreetbets; each node represents a user who submits a post or a comment on the subreddit, and each directed link (*u*, *v*) represents user *u* commenting on a submission by user *v*. The direction of the link represents an interaction, and it is opposite to the information flow (user *u* should have read what *v* wrote to answer, but it is not guaranteed that *v* will read *u*’s reply). Considering all nodes and interactions on WSB between authors discussing about GME, the resulting networked components consist of *N* = 450 710 nodes and *E* = 2 649 108 directed links, activated over the entire period starting on 27 November 2020 and concluding on 3 February 2021.

The time at which posts and comments are published can be used to obtain a description of social interaction dynamics. We modelled such dynamic through temporal *slicing*. In particular, we considered a rolling time window of 7 days and shift it by 2 hours throughout the whole timespan of our dataset, for a total of 1092 windows. For each time window, we constructed a network using posts and comments published during that time window. We tested alternative temporal slicing strategies, and discussed them in electronic supplementary material, §A.6 as well as different sizes of the window with which networks are built in electronic supplementary material, §A.7.

For each slice, we characterized nodes with a number of features, including their age (the time elapsed since their first interaction within the community), in- or out-degree (number of incoming or outgoing edges), their commitment (number of commitment events) or the reach of their commitment (number of users who comment on their commitment events). We also ran *D*_*k*,*l*_-core decomposition [28,41] on the network of each temporal slice. The algorithm partitions nodes by their *core shell* (or core number), i.e. the shell *k*, *l*, defined as the maximal subgraph in which every vertex has at least out-degree *k* and in-degree *l*. The *D*_*k*,*l*_-core decomposition algorithm takes edge directionality into account, but also provides a wider space of cores to explore. To this extent, in the main manuscript, we explore the case in which *D*_*k*,*l*=*k*_ while in the electronic supplementary material, we complement the analysis by providing the results for *D*_*k*,0_, *D*_*k*,1_, *D*_0,*l*_ and *D*_1,*l*_ (see electronic supplementary material, §A.8).

#### 4.4.1. Null model for random commitment activity

When computing the commitment of nodes as a function of their core number, to assess if committed users are more central or peripheral in the network, it is important to compare with a null model which takes into account the network’s topology. For this reason, we consider a null model of random commitment in which committed events are reshuffled randomly over the whole network, while the network’s structure is preserved. The empirical commitment of nodes with core number *k* is then compared with a uniform distribution of commitment across nodes, which is equivalent to averaging the results over an infinite number of random shuffles.

## Data Availability

All data used in this work are publicly available on Reddit and can be accessed via the Reddit API service (https://www.reddit.com/wiki/api-terms). Code and processed data to reproduce figures are made available at https://doi.org/10.5281/zenodo.5783894.
